# The SPECIES and ORGANISMS Resources for Fast and Accurate Identification of Taxonomic Names in Text

**DOI:** 10.1371/journal.pone.0065390

**Published:** 2013-06-18

**Authors:** Evangelos Pafilis, Sune P. Frankild, Lucia Fanini, Sarah Faulwetter, Christina Pavloudi, Aikaterini Vasileiadou, Christos Arvanitidis, Lars Juhl Jensen

**Affiliations:** 1 Institute of Marine Biology, Biotechnology and Aquaculture (IMBBC), Hellenic Centre for Marine Research (HCMR), Heraklion, Greece; 2 Department of Disease Systems Biology, Novo Nordisk Foundation Center for Protein Research, Faculty of Health Sciences, University of Copenhagen, Copenhagen, Denmark; Université Paris-Sud, France

## Abstract

The exponential growth of the biomedical literature is making the need for efficient, accurate text-mining tools increasingly clear. The identification of named biological entities in text is a central and difficult task. We have developed an efficient algorithm and implementation of a dictionary-based approach to named entity recognition, which we here use to identify names of species and other taxa in text. The tool, SPECIES, is more than an order of magnitude faster and as accurate as existing tools. The precision and recall was assessed both on an existing gold-standard corpus and on a new corpus of 800 abstracts, which were manually annotated after the development of the tool. The corpus comprises abstracts from journals selected to represent many taxonomic groups, which gives insights into which types of organism names are hard to detect and which are easy. Finally, we have tagged organism names in the entire Medline database and developed a web resource, ORGANISMS, that makes the results accessible to the broad community of biologists. The SPECIES software is open source and can be downloaded from http://species.jensenlab.org along with dictionary files and the manually annotated gold-standard corpus. The ORGANISMS web resource can be found at http://organisms.jensenlab.org.

## Introduction

We are living in an era in which the paradigm of scientific knowledge communication is shifting [1], [2]. Due to the exponential growth of the biomedical literature, it has become impossible for researchers to read all relevant papers, even on a specialized topic, and text-mining tools are thus becoming essential [3]. Moreover, the fraction of new papers that is published under Open Access licenses is rapidly increasing, making it possible to mine full-text papers rather than only abstracts. Consequently, developing software tools to mine the biomedical literature is challenging both in terms of the *quality* required for the results to be useful to researchers and the *quantity* of text that needs to be processed.

Accurately identifying the taxa mentioned in a document is an important task, both in its own right to classify and retrieve documents, as a prerequisite for resolving inter-species ambiguities when identifying gene and protein names [4], [5], and as a component in natural language systems [6]. The recognition of organism names in scientific documents has been the subject of intensive research, which was summarized well by Gerner et al. [6]. Most of the systems that have been developed fall into two different broad classes, namely rule-based and dictionary-based approaches.

Rule-based systems such as TaxonGrab [7] and FAT [8] exploit the structured form of the Linnaean binomial nomenclature for species names [9] to recognize them in text. Such systems have the advantage that they can recognize new Linnaean names in text without requiring any updates, for which reason they have been used, for example, to process the biodiversity literature [7], [8]. However, rule-based approaches have difficulties with alternative forms of species names such as common (vernacular) names, which are frequently used to refer to model organisms in the biomedical literature [6].

By contrast, dictionary-based systems such as AliBaba [10], Whatizit [11], LINNAEUS [6], and OrganismTagger [12] are equally well suited for recognizing all types of organism names. Another crucial advantage of dictionary-based approaches over rule-based ones is that they are not only able to recognize names in text but also to map them to unique database identifiers [3]. However, they require a comprehensive dictionary of organism names, which must be kept up to date as new names are introduced into the literature, and the entire corpus needs to be re-processed whenever the dictionary is updated. Speed is thus particularly important for dictionary-based systems.

Among the aforementioned systems, LINNAEUS has been employed as a component in many text-mining systems participating in community challenges [5]. This is probably partly because it is available as a command line tool and partly because it has high accuracy [6]. The latter is achieved through a the use of a comprehensive dictionary derived from NCBI Taxonomy to deal with synonyms, a stop-word list to filter out matches to common English words, and post-processing rules to disambiguate homonyms [6]. However, running LINNAEUS on very large corpora takes considerable time.

Assessing how well a system scales to the challenge of the *quantity* of scientific literature and taxonomic names is a simple matter of benchmarking the speed with which it processes documents and its memory requirement. Evaluating the *quality* of organism name identification is less trivial, as it requires a manually annotated corpus that can be used as a gold standard. One such corpus is Linnaeus 100 (L100), which consists of 100 full-text articles in which organism names have been manually annotated [6]. The annotation quality of the L100 corpus is high, for which reason many groups developing organism taggers have used and modified it [12]. However, because it is based on full-text papers, it has low diversity of organism names and high degree of repetition, for which reason it is easy to inadvertently overfit methods – including our own – to this specific corpus and consequently overestimate their accuracy.

Here we present a new open-source software tool for tagging organism names in text, which is more than an order of magnitude faster and much more memory efficient than the commonly used LINNAEUS tagger. The software includes two executables, SPECIES and ORGANISMS, which tag species names and organism names from any taxonomic level, respectively. We also describe a new corpus for benchmarking taggers at the species-level, and show that SPECIES has approximately the same precision and recall as the LINNAEUS tagger, despite being much faster. Finally, we present a search interface that allows researchers to retrieve Medline abstracts about a taxon of interest based on precomputed results from the ORGANISMS tagger.

## Results and Discussion

### Efficient species tagger

We have developed two command-line tools, SPECIES and ORGANISMS, for recognition of organism names in text. To allow for both identification of names in the text and normalization of them to the corresponding entries in the NCBI taxonomy database, we have taken a dictionary-based approach.

The starting point for the dictionary is all names from the NCBI Taxonomy itself, which besides the Linnaean binomial names (e.g. *Cannabis sativa*) contains common names and other synonyms (e.g. hemp and marijuana). We expand the dictionary with automatically generated variants, in particular abbreviated Linnaean names (e.g. *C. sativa*).

The software loads the entire expanded dictionary into a hash table to allow fast lookup of names. The hash table makes use of custom hash and string compare functions to allow for orthographic variation in how the names are written. These functions are case insensitive and further allows for arbitrary insertion or deletion of spaces and hyphens in the names and punctuation characters before or after the names. The latter is important for matching common names that may be written in one word, hyphenated, or in two words (e.g. zebrafish, zebra-fish, and zebra fish). It is also crucial when mining full-text papers in which names may be hyphenated due to line breaks.

The comprehensive dictionary, expansion rules, and flexible matching improve recall at the price of false positives. One cause of false positives is that species names may coincide with, for example, common English words or medical terms. We use a regular expression and a manually curated list to eliminate names that cause many false positives (see Methods). We also automatically detect acronyms defined in the text and resolve them to the long form (see Methods).

An additional problem is that a name may refer to multiple species. This is particularly true for abbreviated names; for example *C. sativa* may refer to *Camelina sativa*, *Cannabis sativa*, or *Castanea sativa*. To disambiguate such cases, we check if other unambiguous names appear in the same document. For example, all mentions of *C. sativa* will be disambiguated to *Cannabis sativa* if either the full Linnaean name or one of its synonyms is mentioned in the same document (see Methods).

**Figure 1:**
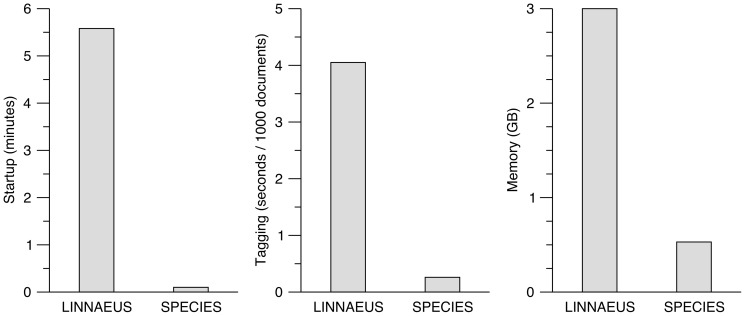
Speed and memory efficiency of the LINNAEUS and SPECIES taggers. The major advantage of the SPECIES tagger over existing methods is its efficiency. Compared to the methodologically similar LINNAEUS tagger, it starts up and loads its dictionary 55× faster (6 seconds vs. 6 minutes 35 seconds), tags Medline abstracts 15× faster (0.26 vs. 4.05 seconds per 1000 documents), and uses 5× less memory in the process (0.5 GB vs. 3.0 GB).

The key strength of our software over existing organism name taggers is its speed and memory efficiency. We compared it to the popular LINNAEUS method, which methodologically is very similar, by processing a set of 536,052 abstracts (Medline 2012, archive files 800–837). Compared to LINNAEUS, the SPECIES software loads its dictionary text files 55× faster, tags documents 15× faster, and uses 5× less memory in the process (Figure 1).

### The Species-800 (S800) corpus

Speed is only one aspect of the performance of named entity recognition system; least as important is the accuracy of the results. To assess this, one needs a gold-standard text corpus, in which mentions of organisms have been manually annotated.

The most commonly used gold standard for organism tagging is the Linnaeus-100 (L100) corpus, which consists of 100 randomly chosen full-text papers. Because L100 is a collection of long documents, it contains few species that are each mentioned many times. This, combined with the fact that L100 was used to guide the development of SPECIES and possibly other methods, gives a high risk of overfitting the methods to this particular corpus.

To better estimate the accuracy of the LINNAEUS and SPECIES methods, we thus developed a new gold-standard corpus called Species-800 (S800). In contrast to L100, S800 is based on abstracts rather than full-text papers; this was decided in order to obtain a more diverse corpus given the same curation effort. To further increase the diversity of species names in the corpus, we constructed S800 by selecting 100 abstracts from journals on each of the following 8 categories: bacteriology, botany, entomology, medicine, mycology, protistology, virology, and zoology (see Methods). This design of the corpus also allows for the accuracy to be assessed separately for each category.

We distributed the 800 abstracts evenly over 5 curators for annotation. The curator guidelines are described in Methods. To ascertain the overall and per-curator quality of annotation, we assigned 20% of abstracts to two curators, who annotated them independently. Based on the shared abstracts we find that the median Cohen's kappa is 0.80, implying that the overall inter-annotator agreement is good (see Table S2 for details).

To put the S800 corpus in perspective we compared it to a corrected version of the L100 corpus (L100E, see Document S1 for details). The S800 corpus contains approximately the same number of annotated species mentions as the L100E corpus. However, because it consists of eight times as many documents from several categories, the diversity of S800 is much higher than that of L100E; the former contains more than three times as many unique species and names as the latter (Table 1).

**Table 1:** Size and species diversity of the corpora.

Corpus	Category	Documents	Unique species	Unique names	Mentions
S800	Protistology	100	196	284	497
	Entomology	100	138	293	614
	Virology	100	117	342	946
	Bacteriology	100	87	179	416
	Zoology	100	85	160	299
	Mycology	100	80	178	538
	Botany	100	68	131	308
	Medicine	100	23	30	90
	Total	800	718	1503	3708
L100E		100	218	375	2988

The number of documents and uniquely annotated species taxonomic ID, unique species names and the number of document level species mentions for the S800 and L100E corpora using the latest version of the NCBI taxonomy.

### Benchmark results

The performance of a named entity recognition method is normally summarized as the precision and recall. However, there are at least two ways to calculate these numbers, because one can count assignments of entities to documents or to individual mentions. Counting at the document level is most relevant if the named entity recognition is used as the basis for information retrieval or document classification, where the logical unit is inherently a document. On the other hand, counting at the mention level is most relevant if the results are to be used as the basis for subsequent information extraction, e.g. as part of an NLP pipeline.

To compare the performance of the LINNAEUS and SPECIES taggers, we have calculated their precision and recall at the document and mention level on the L100E and S800 corpora (Table 2). The SPECIES tagger performs better than the LINNAEUS tagger on the L100E corpus, especially when counting at the mention level. This, however, is unsurprising because we have used the L100E corpus during the development SPECIES, for which reason it may be overfitted on this particular corpus. When the two taggers are instead compared on the new and more diverse S800 corpus, which did not yet exist when either tagger was made, we obtain lower but practically identical performance numbers for the two taggers. Using the updated version of the dictionary that is distributed with the SPECIES tagger leads to unchanged precision and approximately 1 percentage point higher recall on the S800 corpus.

**Table 2:** Summary benchmark of LINNAEUS and SPECIES.

Corpus	Level	Software	Precision	Recall	F1
S800	Document	LINNAEUS	86.4%	89.3%	87.9%
		SPECIES	85.9%	89.8%	87.8%
	Mention	LINNAEUS	84.3%	75.4%	79.6%
		SPECIES	83.9%	72.6%	77.8%
L100E	Document	LINNAEUS	89.2%	91.4%	90.3%
		SPECIES	89.9%	94.3%	92.0%
	Mention	LINNAEUS	88.7%	81.8%	85.1%
		SPECIES	91.5%	90.8%	91.1%

We compared LINNAEUS and SPECIES taggers by calculating their precision and recall on two different corpora (L100E an S800) at the document and at the mention level.

Unsurprisingly, SPECIES performs better than LINNAEUS on the L100E corpus, which we used during the development SPECIES. On the S800 corpus, which did not exist when either tagger was developed, we obtain very similar performance numbers for the two taggers.

**Figure 2:**
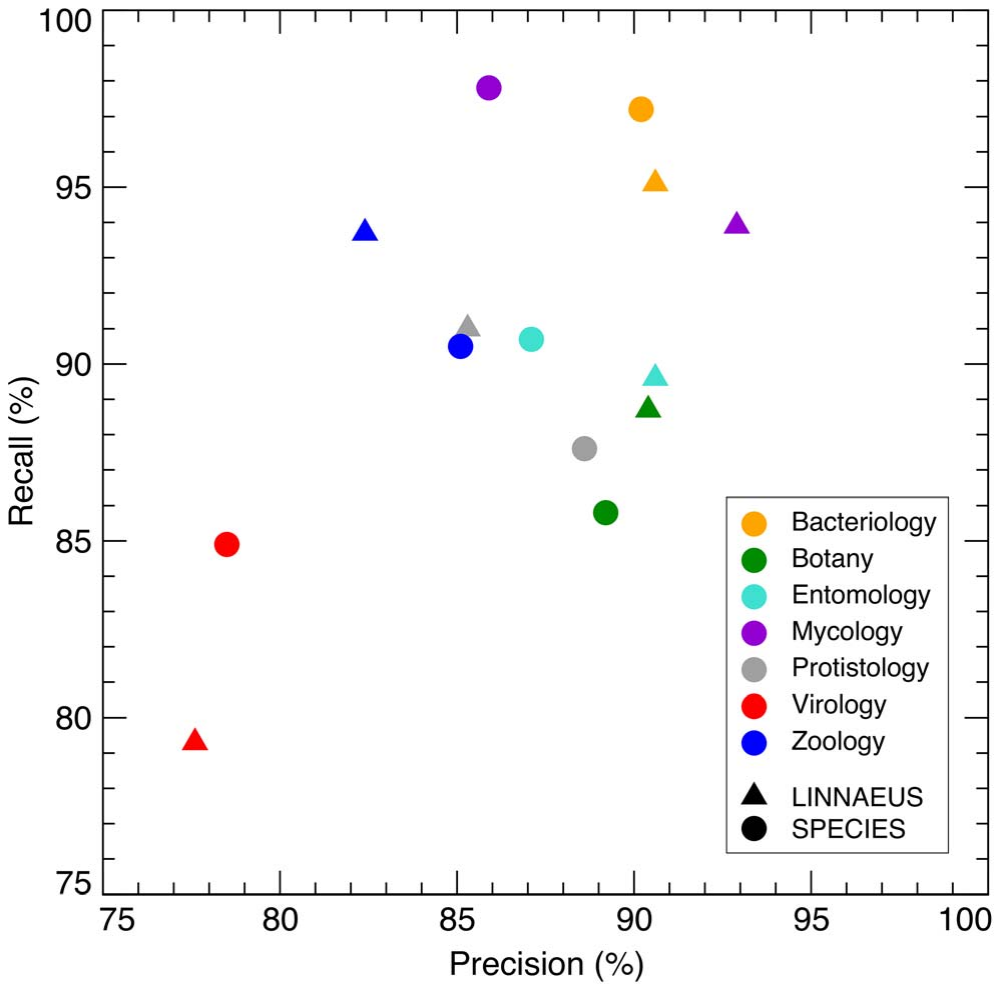
Precision and recall for separate S800 categories. Because the S800 corpus consists of seven different taxonomic categories (the eighth category is not taxonomic), it can provide insights into which types of species are hard to identify in text and which are easy. Plotting the precision and recall on each of the seven categories separately for both the LINNAEUS and the SPECIES tagger shows little difference between the taggers, but big differences between categories. It is clear that both methods are considerably worse at tagging names of viruses than at tagging cellular organisms, and that bacterial and fungal species—for which Linnaean nomenclature is primarily used—are the easiest to identify in text.

The design of the S800 corpus allows us to look into the performance of the LINNAEUS and SPECIES taggers in more detail, by benchmarking their performance separately for different taxonomic groups. This analysis shows that the LINNAEUS and SPECIES taggers give very similar precision and recall not only on the corpus as a whole but also within each category (Figure 2). However, it is clear that some taxonomic categories are inherently more difficult to tag correctly. Both methods perform considerably worse on virology-related abstracts compared to all other categories. Conversely, bacteriology and mycology abstracts are the easiest to tag. This is likely because the binomial Linnaean names are predominantly used in abstracts describing bacteria and fungi, whereas the naming convention for viruses is not as systematic, which causes authors to often deviate from the standardized names.

### The ORGANISMS web resource

The open-source tagging software enables users to quickly and easily tag species names and other organism names in text corpora of any size. We intend the primary users to be other computational researchers who use it as a component in larger text-mining pipelines or workflows.

To make the tool useful to biologists and ecologists as well, we developed the ORGANISMS web resource, which provides access to the tagging results of all abstracts from the Medline database, including all taxonomic levels (as opposed to only species). The resulting database currently contains 23,468,559 matches for 164,084 different taxa in 6,642,192 Medline abstracts.

**Figure 3:**
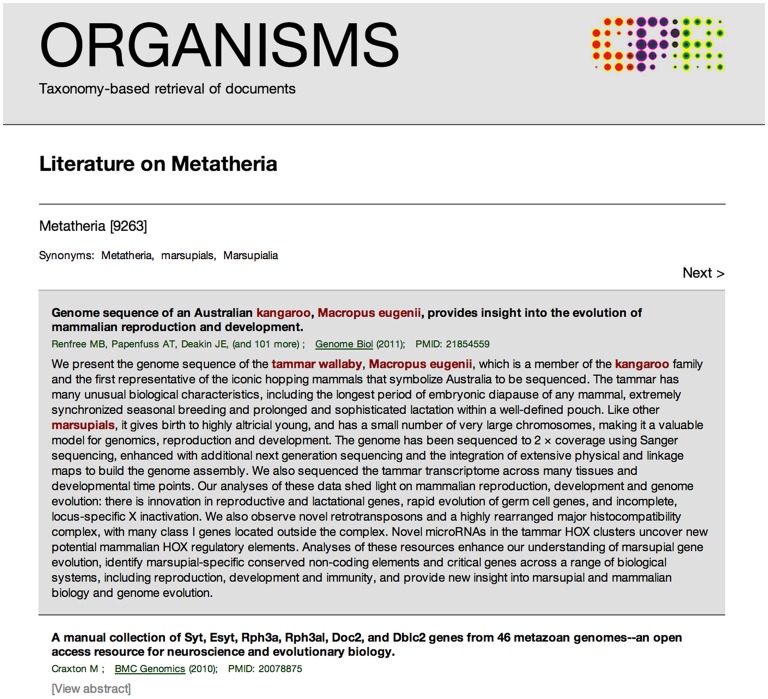
The ORGANISMS web resource. The ORGANISMS web resource (http://organisms.jensenlab.org) aims to make the results of mining the biomedical literature for taxonomic names easily accessible to biologists. It currently covers 164,084 different taxa that can be queried by name. The screenshot shows an example of what is retrieved when searching for Metatheria; because the system is aware of synonyms as well as taxonomy, it correctly retrieved and tagged an abstract about the tammar wallaby.

The simple web interface enables the user to query for any organism from the NCBI taxonomy and view the corresponding abstracts with highlighting of the relevant organism names. Because the underlying tagger takes into account both synonyms and the hierarchical structure of the taxonomy, a search for Metatheria (marsupials) will retrieve both abstracts that explicitly mention the taxon and all abstracts that mention taxa within it, e.g. the tammar wallaby (Figure 3). This makes it particularly useful for finding literature on less studied taxonomic groups.

## Conclusions

We have developed three new freely available resources related to identification of species names in text. First and foremost, we have developed the open source SPECIES tagger, which is many times faster than the commonly used LINNAEUS tagger and matches its good precision and recall. Second, we have created a new gold standard corpus, Species-800, which has been designed to have high diversity of species names. The categorization of the documents within this corpus gives insights into which types of organisms currently present the greatest challenge to text-mining tools. Finally, we provide a web resource that enables the broad community of biologists to make use of the tagging results to retrieve documents that mention any organism of interest.

## Materials and Methods

### The species tagger

#### Dictionary creation and orthographic expansion

The initial dictionary for the SPECIES tagger is based on the complete database dumps of the NCBI Taxonomy (nodes.dmp and names.dmp). Names of higher taxonomic levels than species (e.g. genus names) were discarded and names of lower taxonomic levels (e.g. strain names) were mapped to the NCBI taxonomic identifier of the corresponding species.

Based on the full Linnaean names (e.g. *Cannabis sativa*) the abbreviated forms were automatically generated (e.g. *C. sativa*). We further expanded the dictionary with a small number of additional names – in particular common names – for major model organisms. After benchmarking the SPECIES tagger, we discovered that results can be further improved by also removing the species name from combined species and strain names; this improvement is included in the distributed version of the software, but not in the benchmark comparing the LINNAEUS and SPECIES taggers.

#### Tagging algorithm with flexible matching

To efficiently handle orthographic variation related to whether a name is written as one word, two words or with a hyphen, SPECIES makes use of custom-made hashing and string-compare functions. The hashing function is based on the djb2 hash but considers uppercase and lowercase characters as equivalent and disregards all white space and punctuation characters. The string-compare function is slightly more restrictive; hyphens and white space characters are only disregarded within names, whereas other punctuation characters, such as quotes and parentheses, are only disregarded at the beginning or end of names. To match a document against the dictionary, we first tokenize the text on white space characters and certain special characters such as slash. We next look up all substrings consisting of up to six tokens to identify the left-most longest matches.

The tagger furthermore makes use of a regular expression to find acronyms that are defined within a document. Specifically, we look for an acronym appearing within parentheses immediately following a series of words with matching initial letters. Whenever acronyms are found, all matches of the acronym in question are translated from their short form to the long form, and the latter is looked up in the dictionary.

In case of ambiguous names, which can refer to multiple species, we make use of other names occurring within the same document to disambiguate them if possible. This is of particular importance for handling abbreviated forms of scientific species names. For example, the name *C. sativa* could equally well refer to *Camelina sativa*, *Cannabis sativa*, or *Castanea sativa*. However, if the document also contains the name *Cannabis sativa* (or marijuana) all occurrences of *C. sativa* will be correctly disambiguated to refer only to *Cannabis sativa*.

#### Exclusion of names causing false positives

Using a plain dictionary approach to tag named entities will result in many false positives. To identify the words that cause these, we tagged all Medline abstracts using the SPECIES tagger with the above-mentioned dictionary. Based on this, we extracted all names occurring more than 2000 times, considering different orthographic variants as different names. For all of these names, we manually inspected abstracts to check if the name in the majority of cases referred to the correct organism or if it had a different meaning, in which case it was added to a block list. It is important to note that the benchmark sets were not used for this purpose.

### Creation of the S800 corpus

#### Selection of abstracts

To make a corpus that covers a diverse selection of organisms, we decided on eight categories of articles, and used ISI Web of Knowledge to identify journals for each of them based on journal scope, impact factor, and the number of publications (Table S1). Seven of the categories represent taxonomic groups; the eighth category (Medicine) was primarily included as a negative control in which not many species names are mentioned. From each category, we randomly selected 100 abstracts published in 2011 or 2012, yielding 800 unique abstracts in total. Retractions, letters to the editor, and abstracts of less than 500 characters were excluded. For ease of use, all UTF-8 characters were converted to equivalent ASCII representations.

#### Curator guidelines

The guidelines to curators were to annotate all substrings, which can meaningfully be identified as referring to a taxon. While the main focus was on annotating species mentions, strings referring to any taxonomic level, (e.g. kingdoms, orders, genera, strains) were also considered. The main guidelines were:

- All document substrings must be evaluated and all mentions including repetitions should be listed in the order of appearance in the text.
- The annotated name types among others should include: Linnaean binomials, common names, strain names, author defined acronyms.
- For each annotated string, curators must record the name as it appeared in text and report the corresponding NCBI Taxonomy database identifier.
- Special cases of adjectives being used to indicate a taxon, misspellings, typographic or other errors and enumerations were indicated as such.
- Taxonomic mentions that did not correspond to an existing NCBI Taxonomy database entry were also indicated.

#### Benchmarking

The LINNAEUS and SPECIES taggers were both benchmarked and compared against the S800 corpus specifically restricting the test to the species level. We mapped taxonomic identifiers corresponding to taxa below the species level (e.g. strains) to their parent species, and ignored taxonomic identifiers from levels above the species level (e.g. genera). The LINNAEUS tagger was run without proxy names, which is most consistent with the curator guidelines.

For the document-level statistics we collect for each document the set of unique taxonomic identifiers from the corpus annotation and from the tagger output. These sets are then compared and identifiers in both sets are counted as true positives, those only in the corpus annotation as false negatives, and those only in the tagger output as false positives.

For the mention-level statistics we used flexible boundary matching of species names, meaning that taggers would receive a true positive if it produced a tag that overlapped with an annotated substring and had the correct assigned taxonomic identifier. For example, if the string “E. coli K12” is annotated in S800 and the tagger matches only the string “E. coli”, it will be counted as a true positive (provided the taxonomic identifier is also correct).

Both taggers in a few cases output multiple taxonomic identifiers for a single match. Because this happens only rarely, we decided to count the match as a true positive provided the correct taxonomic identifier was among the suggested ones. Allowing for this flexibility had almost no impact on the precision and recall.

## Supporting Information

Document S1 The SPECIES and ORGANIMS software documentation including library dependencies, an example of how to run the executables, and the description of the output file format.
(DOC)

Table S1 **Journal selection for the S800 categories.** The table provides an overview of the journal selection for the eight categories that make up S800. For each category we selected between one and four journals, from which we randomly picked 100 Medline abstracts in total from the years 2011 and 2012.
(DOC)

Table S2 **Inter-annotator agreement for the S800 corpus.** We quantified the Inter-Annotator-Agreement (IAA) by calculating Cohen's kappa for all pairs of the five curators. Cohen's kappa is defined as kappa = (Po−Pe)/(1-Pe). Po refers the observed probability of agreement between two curators, whereas Pe is the expected probability of agreement by random chance.
(DOC)
