# Supplementary material for: The SPECIES and ORGANISMS Resources for Fast and Accurate Identification of Taxonomic Names in Text
Source: PLoS One. 2013 Jun 18;8(6):e65390. doi: 10.1371/journal.pone.0065390 (PMC3688812; doi:10.1371/journal.pone.0065390)
Supplement: Document S1 — The SPECIES and ORGANIMS software documentation including library dependencies, an example of how to run the executables, and the description of the output file format. (DOC) [file pone.0065390.s001.doc]

**Supplementary Document S1**

## Supplementary Software Documentation

### Dependencies required for compiling SPECIES, ORGANISMS

The SPECIES, ORGANISMS software are coded in C++. In addition to the libraries that are part of any C++ installation, it makes use of two extensions: the C++ Technical Report 1 (TR1) library extensions and the BOOST library.

### Running the SPECIES, ORGANISMS executable

To execute the SPECIES, ORGANISMS taggers, simply run the binary executable obtained from compiling the source code with one command-line argument:

./species *documents_directory* > *matches_file*

./organisms *documents_directory* > *matches_file*

The documents_directory should simply contain each document to be tagged in a separate file. These files must have the extension .txt.

### Output file format

The output of running the SPECIES, ORGANISMS tagger is a tab-delimited file with 5 columns: the name of the document file, the byte position of the first character in the match, the byte position of the last character of the match, the string matched, and the corresponding taxonomic identifier. Each line specifies one match; in case of ambiguity, each possible taxonomic identifier will be listed as a separate line (with the other columns unchanged).
