# Supplementary material for: The SPECIES and ORGANISMS Resources for Fast and Accurate Identification of Taxonomic Names in Text
Source: PLoS One. 2013 Jun 18;8(6):e65390. doi: 10.1371/journal.pone.0065390 (PMC3688812; doi:10.1371/journal.pone.0065390)
Supplement: Table S1 — Journal selection for the S800 categories. The table provides an overview of the journal selection for the eight categories that make up S800. For each category we selected between one and four journals, from which we randomly picked 100 Medline abstracts in total from the years 2011 and 2012. (DOC) [file pone.0065390.s002.doc]

### Supplementary Table S1: Journal selection for the S800 categories

The table provides an overview of the journal selection for the eight categories that make up S800. For each category we selected between one and four journals, from which we randomly picked 100 Medline abstracts in total from the years 2011 and 2012.

| Category | Journal | Abstracts |
| --- | --- | --- |
| Bacteriology | Journal of Bacteriology | 100 |
| Botany | New Phytologist | 50 |
|  | Plant Cell & Environment | 50 |
| Entomology | Insect Molecular Biology | 59 |
|  | Journal Insect Science | 24 |
|  | Environmental Entomology | 17 |
| Medicine | The Lancet | 41 |
|  | The Lancet Infectious Diseases | 15 |
|  | The Lancet Neurology | 22 |
|  | The Lancet Oncology | 22 |
| Mycology | Fungal Genetics and Biology | 100 |
| Protistology | European Journal of Protistology | 30 |
|  | International Journal of Systematic and Evolutionary Microbiology | 30 |
|  | Protist | 40 |
| Virology | Journal of Virology | 63 |
|  | Journal of Virological Methods | 37 |
| Zoology | Journal of Animal Ecology | 100 |
