# Supplementary material for: The SPECIES and ORGANISMS Resources for Fast and Accurate Identification of Taxonomic Names in Text
Source: PLoS One. 2013 Jun 18;8(6):e65390. doi: 10.1371/journal.pone.0065390 (PMC3688812; doi:10.1371/journal.pone.0065390)
Supplement: Table S2 — Inter-annotator agreement for the S800 corpus. We quantified the Inter-Annotator-Agreement (IAA) by calculating Cohen's kappa for all pairs of the five curators. Cohen's kappa is defined as kappa = (Po−Pe)/(1-Pe). Po refers the observed probability of agreement between two curators, whereas Pe is the expected probability of agreement by random chance. (DOC) [file pone.0065390.s003.doc]

### Supplementary Table S2: Inter-annotator agreement for the S800 corpus

We quantified the Inter-Annotator-Agreement (IAA) by calculating Cohen’s kappa for all pairs of the five curators. Cohen’s kappa is defined as kappa = (Po – Pe)/(1-Pe). Po refers the observed probability of agreement between two curators, whereas Pe is the expected probability of agreement by random chance.

| Annotator 1 | Annotator 2 | Cohen’s kappa | Po | Pe |
| --- | --- | --- | --- | --- |
| Christina | Evangelos | 0.86 | 0.870 | 0.038 |
| Christina | Katarina | 0.80 | 0.815 | 0.056 |
| Christina | Lucia | 0.68 | 0.699 | 0.048 |
| Christina | Sarah | 0.63 | 0.643 | 0.041 |
| Evangelos | Katarina | 0.92 | 0.923 | 0.089 |
| Evangelos | Lucia | 0.75 | 0.761 | 0.061 |
| Evangelos | Sarah | 0.80 | 0.810 | 0.046 |
| Katarina | Lucia | 0.79 | 0.803 | 0.058 |
| Katarina | Sarah | 0.78 | 0.800 | 0.078 |
| Lucia | Sarah | 0.86 | 0.863 | 0.044 |
